# 1-(3,5-Dimethyl­phenyl)-2-(4-fluoro­phenyl)-4,5-dimethyl-1*H*-imidazole

**DOI:** 10.1107/S1600536811012098

**Published:** 2011-04-07

**Authors:** S. Rosepriya, A. Thiruvalluvar, J. Jayabharathi, N. Srinivasan, R. J. Butcher, J. P. Jasinski, J. A. Golen

**Affiliations:** a PG Research Department of Physics, Rajah Serfoji Government College (Autonomous), Thanjavur 613 005, Tamilnadu, India; b Department of Chemistry, Annamalai University, Annamalai Nagar 608 002, Tamilnadu, India; c Department of Chemistry, Howard University, 525 College Street NW, Washington, DC 20059, USA; d Department of Chemistry, Keene State College, 229 Main Street, Keene, NH 03435-2001, USA

## Abstract

In the title compound, C_19_H_19_FN_2_, the imidazole ring is essentially planar [maximum deviation of 0.0015 (9) Å] and makes dihedral angles of 77.61 (9) and 26.93 (10)° with the benzene rings attached to nitro­gen and carbon, respectively. The dihedral angle between the two benzene rings is 78.84 (8)°. A C—H⋯π inter­action is found in the crystal structure.

## Related literature

For related structures and applications of imidazole derivatives, see: Gayathri *et al.* (2010 ▶); Rosepriya *et al.* (2011 ▶).
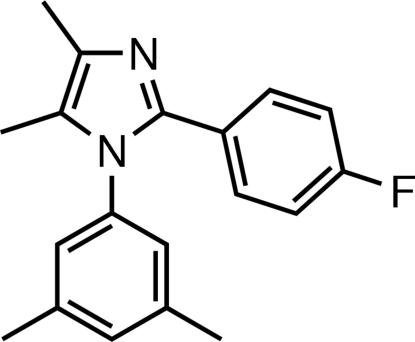


## Experimental

### 

#### Crystal data

C_19_H_19_FN_2_
*M* *_r_* = 294.36
Triclinic, 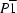

*a* = 8.4226 (10) Å
*b* = 9.5572 (10) Å
*c* = 11.0351 (11) Å
α = 105.423 (9)°
β = 105.677 (9)°
γ = 95.781 (9)°
*V* = 810.07 (17) Å^3^
*Z* = 2
Cu *K*α radiation
μ = 0.63 mm^−1^
*T* = 170 K
0.25 × 0.20 × 0.15 mm

#### Data collection

Oxford Diffraction Xcalibur Eos Gemini diffractometer
Absorption correction: multi-scan (*CrysAlis RED*; Oxford Diffraction, 2010 ▶) *T*_min_ = 0.858, *T*_max_ = 0.911
5121 measured reflections
3054 independent reflections
2771 reflections with *I* > 2σ(*I*)
*R*_int_ = 0.011

#### Refinement

*R*[*F* ^2^ > 2σ(*F* ^2^)] = 0.052
*wR*(*F* ^2^) = 0.159
*S* = 1.07
3054 reflections
203 parameters
H-atom parameters constrained
Δρ_max_ = 0.31 e Å^−3^
Δρ_min_ = −0.23 e Å^−3^

### 

Data collection: *CrysAlis PRO* (Oxford Diffraction, 2010 ▶); cell refinement: *CrysAlis PRO*; data reduction: *CrysAlis RED* (Oxford Diffraction, 2010 ▶); program(s) used to solve structure: *SHELXS97* (Sheldrick, 2008 ▶); program(s) used to refine structure: *SHELXL97* (Sheldrick, 2008 ▶); molecular graphics: *ORTEP-3* (Farrugia, 1997 ▶); software used to prepare material for publication: *PLATON* (Spek, 2009 ▶).

## Supplementary Material

Crystal structure: contains datablocks global, I. DOI: 10.1107/S1600536811012098/hg5018sup1.cif

Structure factors: contains datablocks I. DOI: 10.1107/S1600536811012098/hg5018Isup2.hkl

Additional supplementary materials:  crystallographic information; 3D view; checkCIF report

## Figures and Tables

**Table 1:** Hydrogen-bond geometry (Å, °) *Cg*3 is the centroid of the C21–C26 ring.

*D*—H⋯*A*	*D*—H	H⋯*A*	*D*⋯*A*	*D*—H⋯*A*
C12—H12⋯*Cg*3^i^	0.95	2.86	3.7969 (19)	169

Symmetry code: (i) 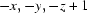.

**Fig. 1.**
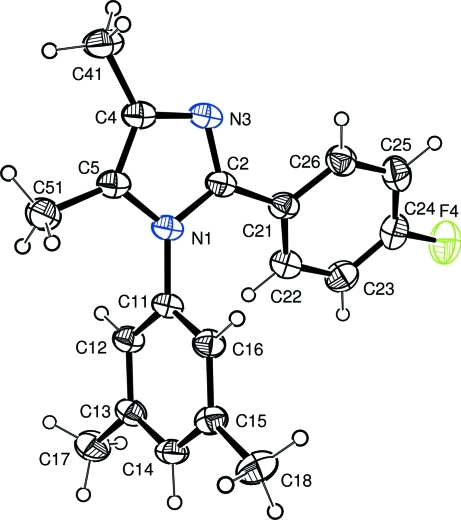
The molecular structure of the title compound, showing the atom-numbering scheme and displacement ellipsoids drawn at the 30% probability level. H atoms are shown as small spheres of arbitrary radius.

## supplementary crystallographic information

### Comment

Rosepriya *et al.* (2011) have reported the crystal structure of 1,2-Diphenyl-1*H*-imidazo[4,5-*f*][1,10]phenanthroline. As part of our research (Gayathri *et al.*, (2010)), we have synthesized the title compound (I) and report its crystal structure here. Since our group doing the research in organic light emitting devices, we are interested to use the title compound as ligand for synthesizing Ir(III) complexes.

In the title compound (Fig. 1), C_19_H_19_FN_2_, the imidazole ring is essentially planar [maximum deviation of 0.0015 (9) Å for C4]. The imidazole ring makes dihedral angles of 77.61 (9) and 26.93 (10)° with the benzene rings attached to N1 and C2, respectively. The dihedral angle between the two benzene rings is 78.84 (8)°. A C12—H12···π interaction involving (C21—C26) ring is found in the crystal structure (Table 1).

### Experimental

To pure butane-2,3-dione (1.48 g, 15 mmol) in ethanol (10 ml), 3,5-xylidine (1.8 g, 15 mmol), ammonium acetate (1.15 g, 15 mmol) and 4-fluorobenzaldehyde (1.7 g, 15 mmol) was added about 1 h by maintaining the temperature at 333 K. The reaction mixture was refluxed for 7 days and extracted with dichloromethane. The solid separated was purified by column chromatography using hexane: ethyl acetate as the eluent. Yield: 2.1 g (48%). Crystals suitable for X-ray diffraction studies were grown by slow solvent evaporation of a solution of the compound in dichloromethane.

### Refinement

H atoms were positioned geometrically and allowed to ride on their parent atoms, with C—H = 0.95 - 0.98 Å; *U*_iso_(H) = k*U*_eq_(C), where k = 1.5 for methyl and 1.2 for all other H atoms.

### Crystal data

**Table:**

C_19_H_19_FN_2_	*Z* = 2
*M**_r_* = 294.36	*F*(000) = 312
Triclinic, *P*1	*D*_x_ = 1.207 Mg m^−^^3^
Hall symbol: -P 1	Melting point: 377 K
*a* = 8.4226 (10) Å	Cu *K*α radiation, λ = 1.54178 Å
*b* = 9.5572 (10) Å	Cell parameters from 3721 reflections
*c* = 11.0351 (11) Å	θ = 5.6–71.2°
α = 105.423 (9)°	µ = 0.63 mm^−^^1^
β = 105.677 (9)°	*T* = 170 K
γ = 95.781 (9)°	Block, colourless
*V* = 810.07 (17) Å^3^	0.25 × 0.20 × 0.15 mm

### Data collection

**Table:**

Oxford Diffraction Xcalibur Eos Gemini diffractometer	3054 independent reflections
Radiation source: Enhance (Cu) X-ray Source	2771 reflections with *I* > 2σ(*I*)
graphite	*R*_int_ = 0.011
Detector resolution: 16.1500 pixels mm^-1^	θ_max_ = 71.3°, θ_min_ = 5.6°
ω scans	*h* = −9→10
Absorption correction: multi-scan (*CrysAlis RED*; Oxford Diffraction, 2010)	*k* = −11→11
*T*_min_ = 0.858, *T*_max_ = 0.911	*l* = −13→10
5121 measured reflections	

### Refinement

**Table:**

Refinement on *F*^2^	Primary atom site location: structure-invariant direct methods
Least-squares matrix: full	Secondary atom site location: difference Fourier map
*R*[*F*^2^ > 2σ(*F*^2^)] = 0.052	Hydrogen site location: inferred from neighbouring sites
*wR*(*F*^2^) = 0.159	H-atom parameters constrained
*S* = 1.07	*w* = 1/[σ^2^(*F*_o_^2^) + (0.0944*P*)^2^ + 0.144*P*] where *P* = (*F*_o_^2^ + 2*F*_c_^2^)/3
3054 reflections	(Δ/σ)_max_ = 0.001
203 parameters	Δρ_max_ = 0.31 e Å^−^^3^
0 restraints	Δρ_min_ = −0.23 e Å^−^^3^

### Special details

**Table:**

Geometry. Bond distances, angles *etc*. have been calculated using the rounded fractional coordinates. All su's are estimated from the variances of the (full) variance-covariance matrix. The cell e.s.d.'s are taken into account in the estimation of distances, angles and torsion angles
Refinement. Refinement of *F*^2^ against ALL reflections. The weighted *R*-factor *wR* and goodness of fit *S* are based on *F*^2^, conventional *R*-factors *R* are based on *F*, with *F* set to zero for negative *F*^2^. The threshold expression of *F*^2^ > 2σ(*F*^2^) is used only for calculating *R*-factors(gt) *etc*. and is not relevant to the choice of reflections for refinement. *R*-factors based on *F*^2^ are statistically about twice as large as those based on *F*, and *R*- factors based on ALL data will be even larger.

### Fractional atomic coordinates and isotropic or equivalent isotropic displacement parameters (Å^2^)

**Table:**

	*x*	*y*	*z*	*U*_iso_*/*U*_eq_	
F4	0.29950 (19)	0.53513 (14)	0.86850 (14)	0.0964 (5)	
N1	0.19985 (15)	−0.06005 (14)	0.37504 (12)	0.0449 (4)	
N3	0.32289 (16)	−0.11576 (15)	0.55605 (13)	0.0520 (4)	
C2	0.27033 (18)	−0.00885 (17)	0.50979 (14)	0.0453 (4)	
C4	0.2859 (2)	−0.23874 (19)	0.44868 (16)	0.0532 (5)	
C5	0.20944 (19)	−0.20827 (17)	0.33550 (15)	0.0488 (5)	
C11	0.14226 (18)	0.02522 (16)	0.28741 (14)	0.0435 (4)	
C12	−0.02859 (18)	0.01651 (18)	0.23336 (15)	0.0480 (5)	
C13	−0.08585 (19)	0.10368 (18)	0.15377 (15)	0.0494 (5)	
C14	0.0323 (2)	0.19566 (17)	0.12941 (14)	0.0515 (5)	
C15	0.2035 (2)	0.20141 (16)	0.17950 (15)	0.0487 (5)	
C16	0.25852 (18)	0.11506 (16)	0.26059 (14)	0.0464 (4)	
C17	−0.2713 (2)	0.0990 (2)	0.0987 (2)	0.0692 (7)	
C18	0.3276 (3)	0.2979 (2)	0.1464 (2)	0.0698 (7)	
C21	0.28022 (18)	0.14031 (17)	0.59540 (15)	0.0472 (5)	
C22	0.1660 (2)	0.2313 (2)	0.56533 (17)	0.0566 (5)	
C23	0.1740 (3)	0.3655 (2)	0.6562 (2)	0.0654 (6)	
C24	0.2952 (3)	0.4060 (2)	0.77740 (19)	0.0660 (6)	
C25	0.4109 (2)	0.3201 (2)	0.81121 (19)	0.0655 (6)	
C26	0.4030 (2)	0.1881 (2)	0.71957 (16)	0.0558 (5)	
C41	0.3314 (3)	−0.3824 (2)	0.4636 (2)	0.0787 (8)	
C51	0.1442 (2)	−0.3012 (2)	0.19482 (17)	0.0623 (6)	
H12	−0.10645	−0.04873	0.25055	0.0575*	
H14	−0.00553	0.25686	0.07653	0.0618*	
H16	0.37514	0.11800	0.29712	0.0556*	
H17A	−0.29302	0.13147	0.01961	0.1037*	
H17B	−0.30948	0.16460	0.16526	0.1037*	
H17C	−0.33197	−0.00229	0.07526	0.1037*	
H18A	0.34608	0.24283	0.06431	0.1046*	
H18B	0.43413	0.32742	0.21839	0.1046*	
H18C	0.28339	0.38610	0.13490	0.1046*	
H22	0.08176	0.20091	0.48163	0.0679*	
H23	0.09729	0.42825	0.63510	0.0784*	
H25	0.49398	0.35114	0.89552	0.0785*	
H26	0.48261	0.12777	0.74092	0.0670*	
H41A	0.45009	−0.38100	0.47068	0.1181*	
H41B	0.26202	−0.46386	0.38633	0.1181*	
H41C	0.31177	−0.39629	0.54360	0.1181*	
H51A	0.14887	−0.40467	0.18925	0.0934*	
H51B	0.21302	−0.26845	0.14515	0.0934*	
H51C	0.02775	−0.29169	0.15728	0.0934*	

### Atomic displacement parameters (Å^2^)

**Table:**

	*U*^11^	*U*^22^	*U*^33^	*U*^12^	*U*^13^	*U*^23^
F4	0.1034 (10)	0.0696 (8)	0.0947 (9)	0.0084 (7)	0.0297 (8)	−0.0060 (7)
N1	0.0417 (6)	0.0541 (7)	0.0451 (7)	0.0131 (5)	0.0125 (5)	0.0248 (5)
N3	0.0508 (7)	0.0622 (8)	0.0497 (7)	0.0169 (6)	0.0121 (6)	0.0291 (6)
C2	0.0399 (7)	0.0568 (9)	0.0451 (7)	0.0108 (6)	0.0128 (6)	0.0250 (6)
C4	0.0533 (9)	0.0573 (9)	0.0561 (9)	0.0180 (7)	0.0150 (7)	0.0279 (7)
C5	0.0466 (8)	0.0547 (9)	0.0516 (8)	0.0152 (6)	0.0156 (6)	0.0243 (7)
C11	0.0430 (7)	0.0527 (8)	0.0410 (7)	0.0148 (6)	0.0131 (6)	0.0222 (6)
C12	0.0418 (8)	0.0586 (9)	0.0494 (8)	0.0121 (6)	0.0143 (6)	0.0250 (7)
C13	0.0469 (8)	0.0581 (9)	0.0445 (8)	0.0182 (7)	0.0102 (6)	0.0187 (7)
C14	0.0634 (10)	0.0533 (9)	0.0448 (8)	0.0218 (7)	0.0150 (7)	0.0243 (7)
C15	0.0567 (9)	0.0479 (8)	0.0496 (8)	0.0139 (6)	0.0223 (7)	0.0206 (6)
C16	0.0418 (7)	0.0533 (8)	0.0490 (8)	0.0134 (6)	0.0155 (6)	0.0207 (6)
C17	0.0519 (10)	0.0793 (12)	0.0756 (12)	0.0238 (9)	0.0056 (8)	0.0327 (10)
C18	0.0764 (12)	0.0665 (11)	0.0864 (13)	0.0152 (9)	0.0385 (11)	0.0419 (10)
C21	0.0437 (7)	0.0569 (9)	0.0481 (8)	0.0075 (6)	0.0178 (6)	0.0248 (7)
C22	0.0565 (9)	0.0630 (10)	0.0535 (9)	0.0151 (7)	0.0163 (7)	0.0224 (8)
C23	0.0670 (11)	0.0609 (10)	0.0762 (12)	0.0192 (8)	0.0287 (9)	0.0248 (9)
C24	0.0680 (11)	0.0561 (10)	0.0684 (11)	−0.0007 (8)	0.0275 (9)	0.0082 (8)
C25	0.0548 (10)	0.0716 (11)	0.0589 (10)	−0.0022 (8)	0.0124 (8)	0.0123 (8)
C26	0.0460 (8)	0.0654 (10)	0.0559 (9)	0.0054 (7)	0.0132 (7)	0.0227 (8)
C41	0.1018 (16)	0.0660 (12)	0.0736 (12)	0.0332 (11)	0.0157 (11)	0.0351 (10)
C51	0.0685 (11)	0.0640 (10)	0.0532 (9)	0.0186 (8)	0.0135 (8)	0.0192 (8)

### Geometric parameters (Å, °)

**Table:**

F4—C24	1.359 (2)	C24—C25	1.375 (3)
N1—C2	1.3713 (19)	C25—C26	1.374 (3)
N1—C5	1.385 (2)	C12—H12	0.9500
N1—C11	1.442 (2)	C14—H14	0.9500
N3—C2	1.322 (2)	C16—H16	0.9500
N3—C4	1.367 (2)	C17—H17A	0.9800
C2—C21	1.466 (2)	C17—H17B	0.9800
C4—C5	1.362 (2)	C17—H17C	0.9800
C4—C41	1.500 (3)	C18—H18A	0.9800
C5—C51	1.486 (2)	C18—H18B	0.9800
C11—C12	1.385 (2)	C18—H18C	0.9800
C11—C16	1.381 (2)	C22—H22	0.9500
C12—C13	1.393 (2)	C23—H23	0.9500
C13—C14	1.389 (2)	C25—H25	0.9500
C13—C17	1.507 (2)	C26—H26	0.9500
C14—C15	1.387 (2)	C41—H41A	0.9800
C15—C16	1.394 (2)	C41—H41B	0.9800
C15—C18	1.506 (3)	C41—H41C	0.9800
C21—C22	1.393 (2)	C51—H51A	0.9800
C21—C26	1.401 (2)	C51—H51B	0.9800
C22—C23	1.385 (3)	C51—H51C	0.9800
C23—C24	1.374 (3)		
			
C2—N1—C5	107.12 (13)	C13—C14—H14	119.00
C2—N1—C11	127.30 (14)	C15—C14—H14	119.00
C5—N1—C11	125.28 (12)	C11—C16—H16	120.00
C2—N3—C4	106.09 (13)	C15—C16—H16	120.00
N1—C2—N3	110.65 (14)	C13—C17—H17A	109.00
N1—C2—C21	126.33 (14)	C13—C17—H17B	109.00
N3—C2—C21	122.95 (13)	C13—C17—H17C	109.00
N3—C4—C5	110.80 (16)	H17A—C17—H17B	110.00
N3—C4—C41	121.02 (15)	H17A—C17—H17C	109.00
C5—C4—C41	128.18 (16)	H17B—C17—H17C	110.00
N1—C5—C4	105.35 (14)	C15—C18—H18A	109.00
N1—C5—C51	122.36 (14)	C15—C18—H18B	109.00
C4—C5—C51	132.29 (16)	C15—C18—H18C	109.00
N1—C11—C12	119.20 (14)	H18A—C18—H18B	109.00
N1—C11—C16	119.31 (14)	H18A—C18—H18C	109.00
C12—C11—C16	121.49 (15)	H18B—C18—H18C	110.00
C11—C12—C13	119.80 (15)	C21—C22—H22	120.00
C12—C13—C14	118.19 (15)	C23—C22—H22	120.00
C12—C13—C17	120.13 (16)	C22—C23—H23	121.00
C14—C13—C17	121.67 (16)	C24—C23—H23	121.00
C13—C14—C15	122.39 (15)	C24—C25—H25	121.00
C14—C15—C16	118.59 (15)	C26—C25—H25	121.00
C14—C15—C18	120.88 (16)	C21—C26—H26	119.00
C16—C15—C18	120.54 (16)	C25—C26—H26	119.00
C11—C16—C15	119.50 (15)	C4—C41—H41A	109.00
C2—C21—C22	123.84 (14)	C4—C41—H41B	109.00
C2—C21—C26	117.57 (15)	C4—C41—H41C	109.00
C22—C21—C26	118.34 (16)	H41A—C41—H41B	109.00
C21—C22—C23	120.67 (17)	H41A—C41—H41C	109.00
C22—C23—C24	118.6 (2)	H41B—C41—H41C	109.00
F4—C24—C23	118.8 (2)	C5—C51—H51A	109.00
F4—C24—C25	118.53 (18)	C5—C51—H51B	109.00
C23—C24—C25	122.71 (19)	C5—C51—H51C	109.00
C24—C25—C26	118.09 (17)	H51A—C51—H51B	109.00
C21—C26—C25	121.54 (17)	H51A—C51—H51C	109.00
C11—C12—H12	120.00	H51B—C51—H51C	109.00
C13—C12—H12	120.00		
			
C5—N1—C2—N3	0.02 (19)	C41—C4—C5—C51	1.4 (3)
C5—N1—C2—C21	−176.74 (15)	N1—C11—C12—C13	176.87 (14)
C11—N1—C2—N3	−173.88 (14)	C16—C11—C12—C13	−2.3 (2)
C11—N1—C2—C21	9.4 (3)	N1—C11—C16—C15	−177.86 (14)
C2—N1—C5—C4	−0.20 (18)	C12—C11—C16—C15	1.3 (2)
C2—N1—C5—C51	179.77 (15)	C11—C12—C13—C14	1.1 (2)
C11—N1—C5—C4	173.87 (15)	C11—C12—C13—C17	−177.75 (15)
C11—N1—C5—C51	−6.2 (2)	C12—C13—C14—C15	1.1 (2)
C2—N1—C11—C12	−105.84 (19)	C17—C13—C14—C15	179.93 (15)
C2—N1—C11—C16	73.4 (2)	C13—C14—C15—C16	−2.1 (2)
C5—N1—C11—C12	81.3 (2)	C13—C14—C15—C18	177.47 (16)
C5—N1—C11—C16	−99.51 (18)	C14—C15—C16—C11	0.9 (2)
C4—N3—C2—N1	0.16 (18)	C18—C15—C16—C11	−178.71 (15)
C4—N3—C2—C21	177.05 (15)	C2—C21—C22—C23	174.03 (18)
C2—N3—C4—C5	−0.3 (2)	C26—C21—C22—C23	0.0 (3)
C2—N3—C4—C41	178.74 (17)	C2—C21—C26—C25	−173.55 (16)
N1—C2—C21—C22	27.5 (3)	C22—C21—C26—C25	0.9 (3)
N1—C2—C21—C26	−158.45 (16)	C21—C22—C23—C24	−1.0 (3)
N3—C2—C21—C22	−148.95 (17)	C22—C23—C24—F4	−177.64 (19)
N3—C2—C21—C26	25.2 (2)	C22—C23—C24—C25	1.3 (3)
N3—C4—C5—N1	0.30 (19)	F4—C24—C25—C26	178.47 (18)
N3—C4—C5—C51	−179.66 (17)	C23—C24—C25—C26	−0.5 (3)
C41—C4—C5—N1	−178.63 (19)	C24—C25—C26—C21	−0.7 (3)

### Hydrogen-bond geometry (Å, °)

**Table:**

Cg3 is the centroid of the C21–C26 ring.

**Table:**

*D*—H···*A*	*D*—H	H···*A*	*D*···*A*	*D*—H···*A*
C12—H12···Cg3^i^	0.95	2.86	3.7969 (19)	169

Symmetry codes: (i) −*x*, −*y*, −*z*+1.
